# Association Between Hepatitis B Surface Antigen Positivity and Tumor Incidence: A Large Prospective Cohort Study in Southern China

**DOI:** 10.3390/cancers18213445

**Published:** 2026-10-25

**Authors:** Qilan Wen, Yu Wang, Yikun Chang, Jinwei Chen, Na Cao, Xin Sun, Yuantao Hao, Wangjian Zhang, Zhicheng Du

**Affiliations:** 1 Department of Medical Statistics, School of Public Health, Sun Yat-sen Global Health Institute, Center for Health Information Research, Sun Yat-sen University, Guangzhou 510080, China; 2 Center for Public Health and Epidemic Preparedness and Response, Peking University Beijing 100191, China; 3 Department of Epidemiology and Biostatistics, School of Public Health, Peking University, Beijing 100191, China; 4 Key Laboratory of Epidemiology of Major Diseases, Peking University, Ministry of Education, Beijing 100191, China; 5 Guangzhou Joint Research Center for Disease Surveillance and Risk Assessment, Sun Yat-sen University, Guangzhou Center for Disease Control and Prevention, Guangzhou 510440, China

**Keywords:** hepatitis B surface antigen, hepatitis B virus, tumor incidence, cancer epidemiology, prospective cohort, cox proportional hazards model

## Abstract

**Background:** Hepatitis B virus (HBV) infection is a major global health concern and has been implicated in carcinogenesis. However, evidence regarding the association between hepatitis B surface antigen (HBsAg) positivity and the risk of overall and site-specific tumors remains limited. This study aimed to investigate the association between HBsAg positivity and tumor incidence in a large prospective cohort. **Methods:** This prospective cohort study included 538,573 participants recruited in southern China between 2009 and 2015. Newly recorded tumor diagnoses were identified by linkage to a hospital discharge database available through December 2022. Missing covariate values were addressed using five multiple imputations, and propensity score matching was performed separately within each dataset. Cox model estimates were combined using Rubin’s rules. Time-varying and landmark analyses were also performed. **Results:** Baseline HBsAg positivity was associated with increased risks of all-site tumors (pooled hazard ratio [HR], 2.02; 95% confidence interval [CI], 1.79–2.27), digestive system malignancies (pooled HR, 4.28; 95% CI, 3.51–5.22), and benign tumors (pooled HR, 1.52; 95% CI, 1.27–1.82). Strong associations were observed for liver and intrahepatic bile duct malignancies (pooled HR, 15.49; 95% CI, 8.99–26.67) and hepatocellular carcinoma (pooled HR, 19.78; 95% CI, 7.19–54.44). In contrast, the association with extrahepatic digestive system malignancies was not statistically significant. The associations generally attenuated over follow-up but persisted after excluding tumor events recorded within the first 1 and 2 years. Stronger associations with all-site tumors were observed among men and participants aged ≥45 years. **Conclusions:** Baseline HBsAg positivity was associated with an increased risk of overall and site-specific tumors, particularly digestive system malignancies. The associations were stronger among men and older adults. These findings support further clinical evaluation of HBsAg-positive individuals and guideline-based liver cancer surveillance when clinically indicated.

## Simple Summary

Hepatitis B virus infection is an established risk factor for liver cancer, but its associations with tumors at other anatomical sites remain unclear. In this large record-linked cohort study in southern China, baseline hepatitis B surface antigen (HBsAg) positivity was associated with increased risks of hospital-recorded all-site tumors and benign tumors. The association with digestive system malignancies was largely driven by liver and intrahepatic bile duct malignancies, particularly hepatocellular carcinoma, whereas no significant association was observed for extrahepatic digestive system malignancies. The associations were generally strongest during the first several years after cohort entry and were stronger among men and participants aged ≥45 years. These findings support further clinical evaluation of HBsAg-positive individuals and guideline-based liver cancer surveillance when indicated.

## 1. Introduction

Malignant tumors represent a major global public health challenge, consistently ranking among the leading causes of death and placing substantial strain on healthcare systems. According to Global Cancer Observatory estimates, there were approximately 19.3 million new cancer cases and nearly 10.0 million cancer deaths worldwide in 2020 [1]. In China, there were over 4.8 million new cancer cases and nearly 2.57 million deaths in 2022 [2], further highlighting the growing challenges in cancer prevention and control. Although benign tumors are typically non-fatal, several types are highly prevalent in the general population and may lead to considerable morbidity, reduced quality of life, and increased healthcare utilization [3]. Given the heterogeneous nature of tumors across anatomical sites and pathological types, identifying factors associated with both overall and site-specific tumor risks is of significant public health importance. Such evidence is essential for informing targeted prevention and screening strategies and optimizing healthcare resource allocation.

Among the various factors associated with tumor development, chronic viral infections have attracted increasing attention due to their substantial contribution to tumor burden worldwide. Hepatitis B virus (HBV) infection remains a major public health challenge, with approximately 254 million people living with chronic HBV infection worldwide in 2022 [4], a large proportion of whom reside in Asia [5]. HBsAg is a key serological marker of current HBV infection and is widely used in population screening. However, a single baseline HBsAg measurement cannot establish infection persistence, duration, or viral activity. Extensive evidence has established chronic HBV infection as a major etiological factor for hepatocellular carcinoma [6,7]. In addition, accumulating evidence suggests that the effects of HBV infection may not be limited to the liver [8]. HBV-associated systemic inflammation and immune dysregulation may also influence extrahepatic tissues, thereby potentially increasing the risk of malignancies at multiple sites, such as digestive system cancers, endocrine-related tumors, and non-Hodgkin lymphoma [9,10,11,12].

Taken together, several important gaps remain in the existing literature. First, although several cohort studies have examined the association between chronic HBV infection and extrahepatic cancers, existing evidence has mainly focused on selected malignancies, and findings across tumor sites and populations have not been fully consistent [9,10,11,12]. Second, few studies have simultaneously evaluated tumor across a broad range of anatomical sites within a single large population-based cohort, and evidence regarding benign tumors remains particularly limited. Third, most prior studies have primarily reported an overall hazard ratio estimated over the entire follow-up period, which may not fully reflect the temporal changes in the association between baseline HBsAg positivity and tumor risk. Whether this association is constant over time or concentrated during specific follow-up windows remains unclear, yet this question has direct implications for risk stratification and monitoring among HBsAg-positive individuals.

To address these gaps, we evaluated the association between HBsAg positivity and the risk of overall and site-specific tumors, including benign tumors, in 538,573 participants from the Pearl River Cohort in southern China with up to 12 years of follow-up.

## 2. Materials and Methods

### 2.1. Study Design and Population

**Figure 1:**
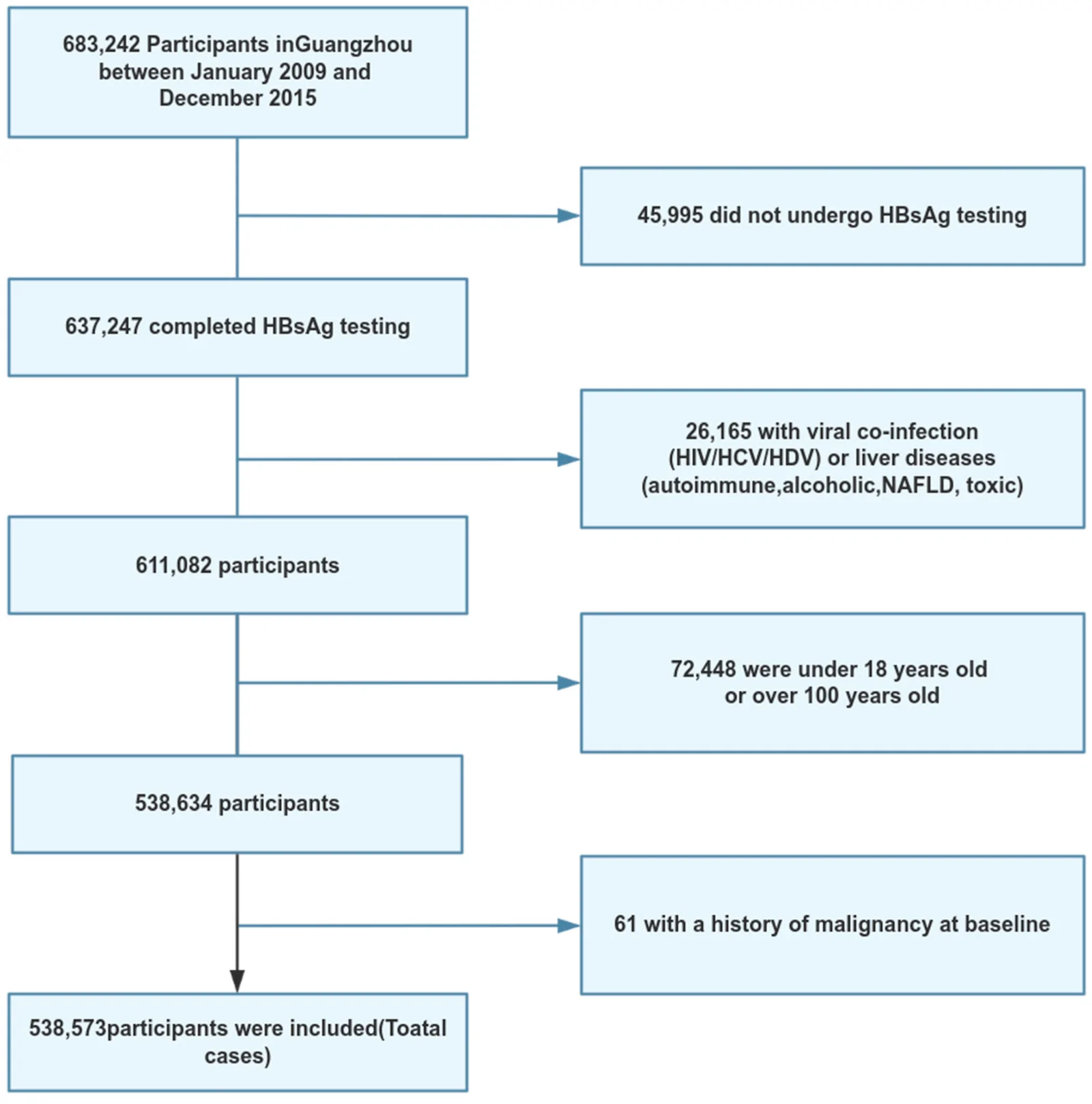
Flow chart of the selection procedure of participants.

The attendees consisted of a part of Major Projects of Science Research for the 11th, 12th and 13th Five-year Plans in China and cohort details were previously published [13]. In this study, we focused on the Guangzhou sub-cohort. Between 2009 and 2015, based on sociodemographic characteristics, we randomly selected 35 communities in Guangzhou and recruited 683,242 eligible participants (permanent residents able to undergo a physical examination). Participants were excluded if they had no available baseline HBsAg measurement, did not meet the prespecified age criteria, had HIV, HCV, or HDV co-infection or other specified liver diseases, or reported a history of tumors at baseline. After sequential application of these criteria, 538,573 participants remained eligible for the study (Figure 1). Newly recorded tumor diagnoses were ascertained by linking baseline records to the hospital discharge abstract database available through December 2022.

Trained nurses conducted standardized face-to-face interviews to collect baseline information. All participants signed informed consent. This investigation was approved by the Human Ethics Committee at Sun Yat-sen University (No. L2017030).

### 2.2. Exposure

The primary exposure variable was the HBsAg status. HBsAg was detected using an enzyme-linked immunosorbent assay kit, performed by a clinical laboratory with standardized quality control (DAAN GENE, Guangzhou, China). Based on the test results, participants were classified as HBsAg positive[HBsAg(+)] or HBsAg negative[HBsAg(−)]. All exposure information was obtained from baseline physical examination records. 

### 2.3. Outcome

The primary outcomes included all-site tumor incidence as well as specific subtypes. During follow-up, participants were linked to the hospital discharge database (front page of medical records) using unique personal identifiers to obtain clinical information and ascertain incident tumor events. We used the International Classification of Diseases, 10th Revision (ICD-10) to identify all-site tumors (C00-C97, D00-D48). For subtypes, we further assessed the five most common tumor types, including: Digestive system malignancies (C15–C26), benign tumors (D10–D36), Respiratory and intrathoracic malignancies (C30–C39), breast cancer (C50), and Thyroid and other endocrine gland malignancies (C73–C75). To further distinguish the contribution of hepatic malignancies, digestive system malignancies were subdivided into liver and intrahepatic bile duct malignancies (C22) and extrahepatic digestive system malignancies (C15–C21 and C23–C26). Hepatocellular carcinoma was separately identified using code C22.0.

Administrative observation time was calculated from the baseline examination to the first hospital-recorded tumor diagnosis or 30 December 2022, whichever occurred first. Participants without a recorded tumor diagnosis were administratively censored at the database cutoff. Because individual-level information on mortality, migration, and loss to follow-up was unavailable, death could not be treated as a verified censoring event or competing event.

### 2.4. Statistical Analysis

Missing values (Table S1, Figure S1) were imputed using multiple imputation by chained equations (MICE) [14] with five imputations. The imputation results showed good convergence, with the distributions of observed and imputed values being consistent. (Figure S2). To control for potential confounding factors between the HBsAg(+) and HBsAg(−) groups, we applied propensity score matching [15] to achieve baseline balance between the groups. Matching was then performed using a 1:1 nearest neighbor method (without replacement), with the caliper set to 0.2 times the standard deviation of the logit of the propensity score [16,17]. Descriptive analyses were based on the first imputed and matched dataset.

Cox proportional hazards models were fitted separately in each of the five imputed and matched datasets. We built three models [18], with Model 3 designated as the primary model and adjusted for potential confounders. For certain tumor subtypes with sparse events, the adjustment set was simplified by excluding covariates that resulted in model non-convergence, to ensure stable estimates [19]. Log-HR estimates and their variances were combined across the five datasets using Rubin’s rules and then exponentiated to obtain pooled HRs and 95% CIs.

We used the Schoenfeld residuals test to assess the proportional hazards assumption for HBsAg [20,21]. For models that did not meet the Proportional Hazards (PH) assumption (Table S2), we built a time-dependent Cox model, adding an interaction term between HBsAg and follow-up time (linear or logarithmic form) [22]. The logarithmic time-dependent Cox model was selected based on AIC (Table S3). Time-varying log-HRs were pooled across the five datasets at each time point using Rubin’s rules. This analysis was also performed separately for participants aged <45 and ≥45 years.

Subgroup analyses were conducted by age, sex, body mass index, educational attainment, marital status, exercise frequency, and smoking status. Effect modification was assessed using interaction terms, with joint tests applied to variables with more than two categories. Interaction estimates were pooled using Rubin’s rules. Ethnicity- and alcohol-stratified results were not reported because of complete separation. We performed sensitivity analyses using alternative covariate categorizations, 1- and 2-year landmark analyses, and comparisons of time-varying estimates across the five imputed datasets. All analyses were performed using R version 4.4.1 with the mice (version 3.18.0), MatchIt (version 4.7.1), and survival (version 3.6.4) packages.

## 3. Results

### 3.1. Baseline Characteristics of Study Participants

Table 1 shows that the study included 538,573 participants, with 117,03 in the HBsAg(+) group and 526,870 in the HBsAg(−) group before matching. After 1:1 propensity score matching, 117,01 matched pairs were included. After matching, the differences between the groups were no longer statistically significant (all *p* > 0.05), suggesting that the matching process effectively balanced the covariates. In the first imputed and propensity score–matched dataset, 23,402 participants contributed 215,648 person-years of administrative observation, with a median observation time of 9.05 years (IQR, 7.79–10.84) (Table S4) Additionally, a description of the entire population was displayed in Table S1.

### 3.2. Distribution Characteristics of Different Tumor Types

**Figure 2:**
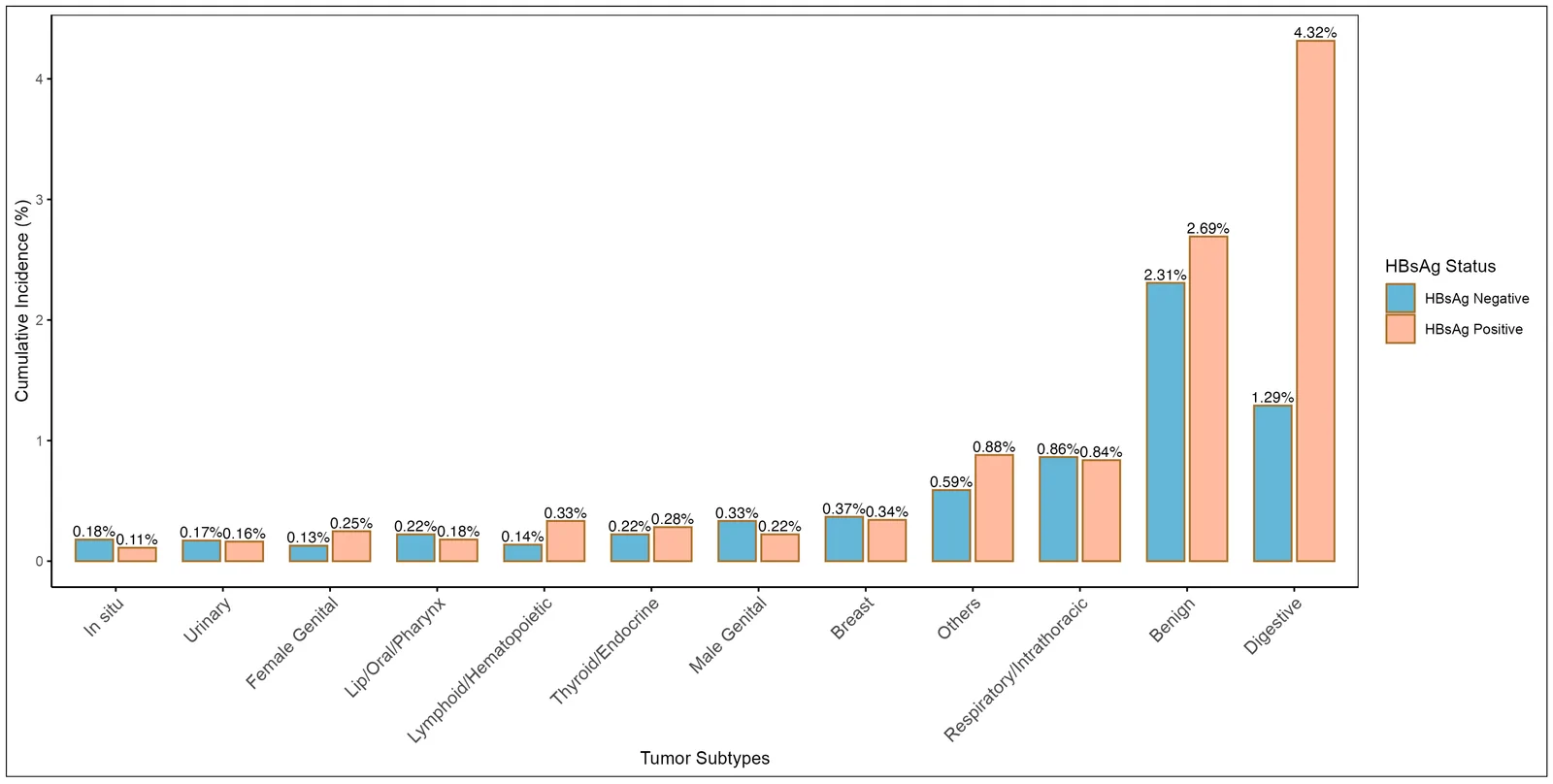
Cumulative incidence of different tumor types in HBsAg-positive and HBsAg-negative groups. The *x*-axis represents tumor types, and the *y*-axis shows cumulative incidence (%). Bars indicate the differences in tumor incidence rates between the HBsAg-positive and HBsAg-negative participants.

Table S5 shows the distribution of different tumor types in the HBsAg(+) and HBsAg(−) groups. Among the HBsAg(+) group, the highest proportion of tumors was observed in the digestive system (4.32%), significantly higher than the 1.29% in the HBsAg(−) group. This was followed by benign tumors (2.69% vs. 2.31%). The distribution of respiratory, breast, and endocrine gland tumors showed relatively minor differences between the two groups. Overall, the differences in tumor type distribution between the two groups were statistically (*χ^2^*, 112.89, *p* < 0.001), indicating a greater tendency for digestive system-related tumors in the HBsAg(+) group (Table S5 and Figure 2).

### 3.3. HBsAg Status and Tumor Risk

After matching, the all-site tumor incidence in the HBsAg(+) group was 10.6% (1241/11,701), higher than the 6.8% (797/11,701) in the HBsAg(−) group. This difference was statistically significant (Risk Ratio, 1.56, 95% confidence interval [CI], 1.43–1.70, *p* < 0.001) (Table S6), suggesting a significant association between HBsAg(+) and increased risk of all-site tumors.

**Figure 3:**
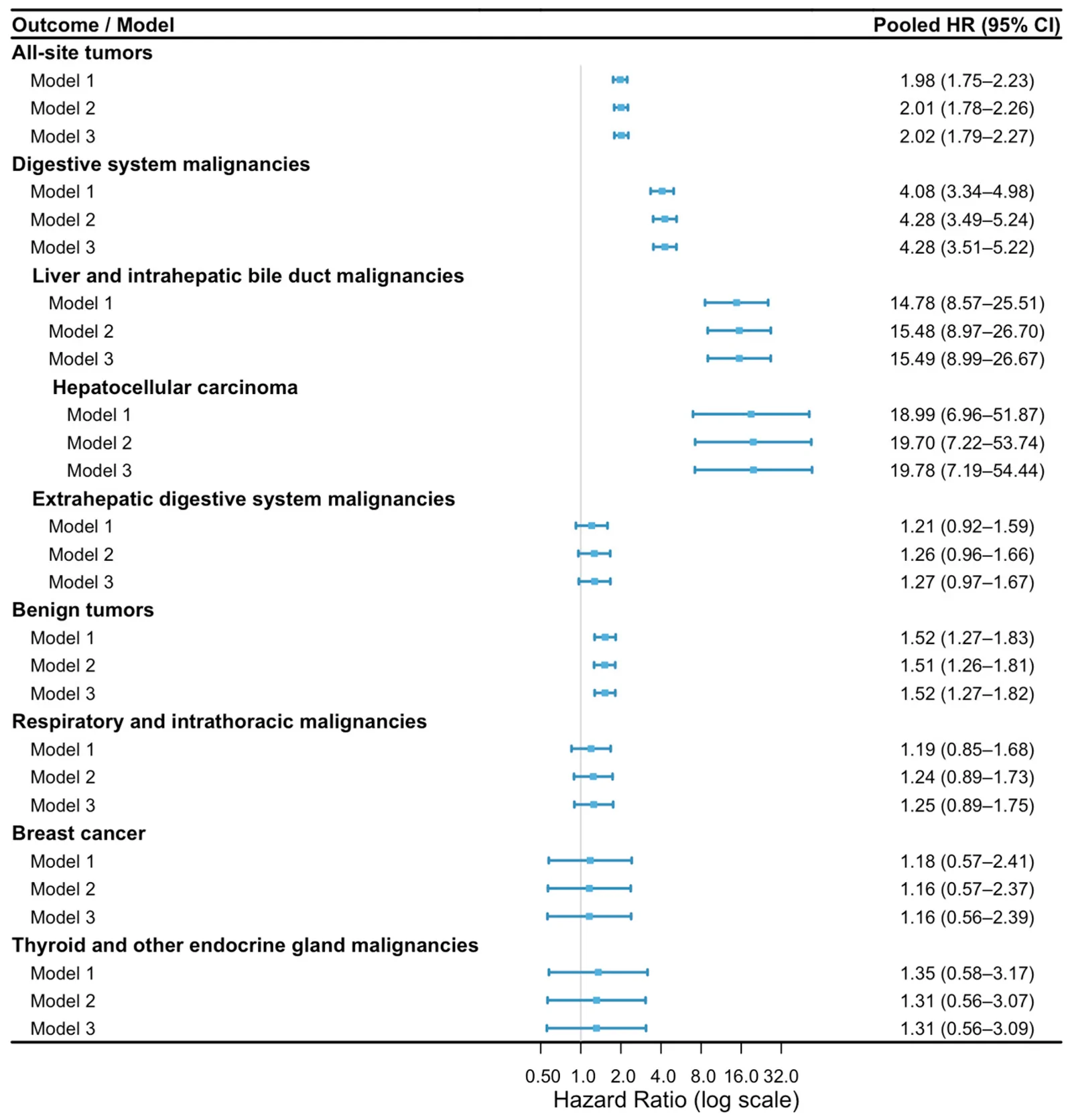
Association between HBsAg positivity and the risk of overall and site-specific tumors under different models. Model 1 represents a traditional Cox proportional hazards model adjusted only for HBsAg exposure. Model 2 adjusts for baseline age and gender in addition to HBsAg exposure. Model 3 further adjusts for ethnicity, smoking status, marital status, education level, exercise frequency, and alcohol consumption. Outcomes included all-site tumors, digestive system malignancies, liver and intrahepatic bile duct malignancies, hepatocellular carcinoma, extrahepatic digestive system malignancies, benign tumors, respiratory and intrathoracic malignancies, breast cancer, and thyroid and other endocrine gland malignancies. Hazard ratios and 95% confidence intervals were pooled across the five imputed datasets using Rubin’s rules. Blue dots represent pooled hazard ratios (HRs), and horizontal blue lines indicate 95% confidence intervals (CIs).

Figure 3 showed the multi-model analysis of the association between HBsAg(+) and the risk of various tumors. Model 3 reveals a significant association between HBsAg(+) and all-site tumor risk, with a pooled hazard ratio[HR] of 2.02 (95% CI, 1.79 –2.27). Among the different tumor types, the greatest increase in risk was observed for digestive system malignancies, with an HR of 4.28 (95% CI, 3.51–5.22) in Model 3, indicating a strong association. Further subdivision of digestive system malignancies showed substantially elevated risks of liver and intrahepatic bile duct malignancies (HR, 15.49; 95% CI, 8.99–26.67) and hepatocellular carcinoma (HR, 19.78; 95% CI, 7.19–54.44). In contrast, the association with extrahepatic digestive system malignancies was not statistically significant (HR, 1.27; 95% CI, 0.97–1.67). For benign tumors, HBsAg(+) also exhibited an increased risk, with an HR of 1.52 (95% CI, 1.27–1.82) in Model 3. In the case of respiratory and thoracic malignancies, breast cancer, and thyroid and other endocrine gland malignancies, HBsAg(+) showed an upward trend, though the results were not statistically significant (95% CI crossing 1). Overall, baseline HBsAg positivity was associated with higher risks of all-site tumors and benign tumors, while the association with digestive system malignancies was predominantly attributable to hepatic malignancies.

**Figure 4:**
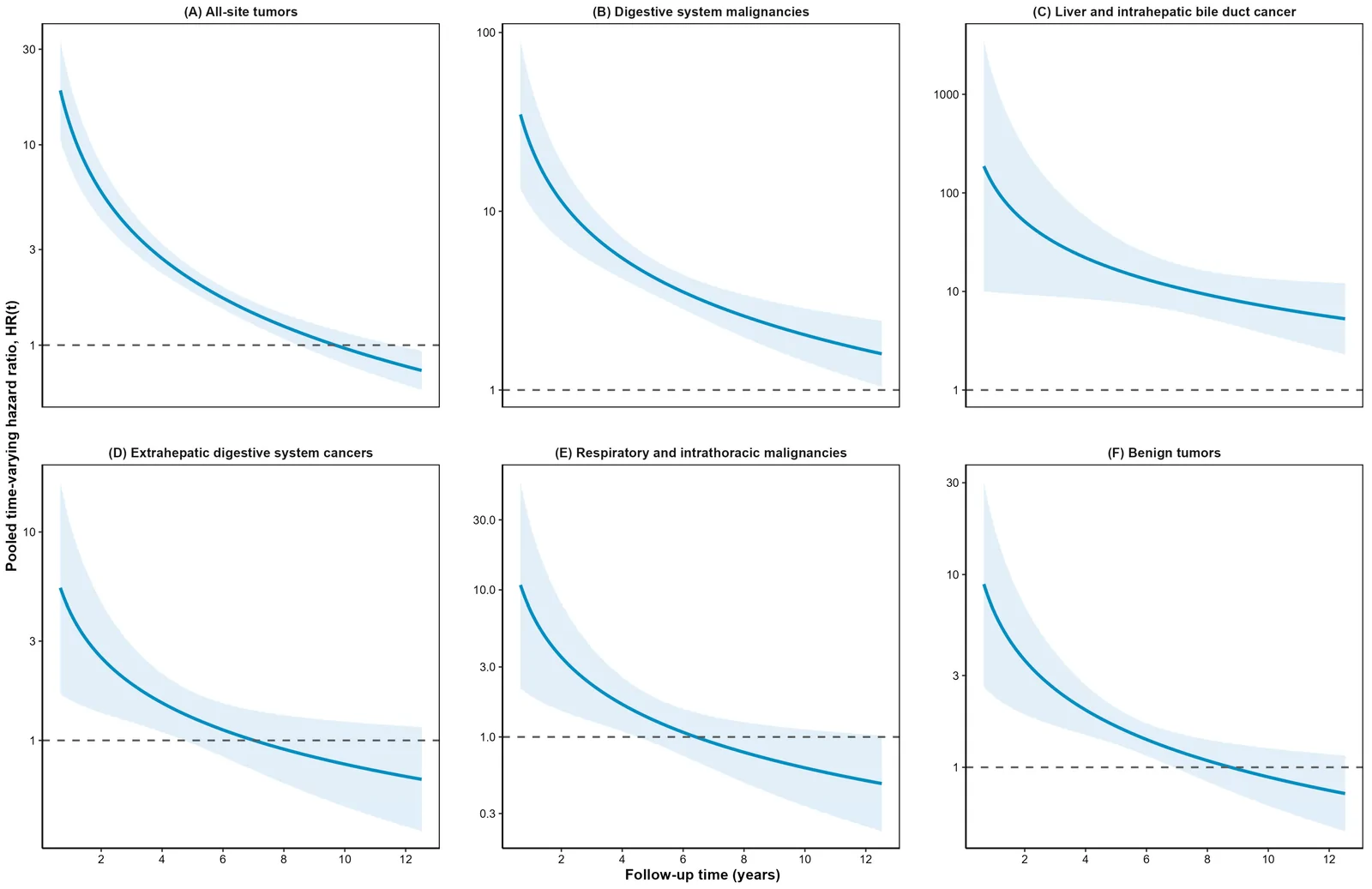
Time-dependent hazard ratios [HRs] and 95% confidence intervals [CIs] for the association between HBsAg positivity and the risk of overall and site-specific tumors in the time-dependent Cox model. The horizontal gray dashed line represents the reference line with HR = 1.

Additionally, Schoenfeld residuals tests indicated that the risk effect of HBsAg changes over time for some tumor outcomes (Table S2). Therefore, we built a Cox model with a time-varying coefficient for HBsAg status. The pooled estimates showed that the associations were strongest during the early years of follow-up (approximately 2–4 years) and generally attenuated over time (Figure 4). For all-site tumors, extrahepatic digestive system cancers, respiratory and intrathoracic malignancies, and benign tumors, the estimated HRs gradually decreased, approaching 1 after 6–10 years. By contrast, the associations with digestive system malignancies and, particularly, liver and intrahepatic bile duct cancer remained elevated throughout the observed follow-up period, although the confidence intervals were wide, especially at the beginning of follow-up. Overall, these findings indicate that the magnitude of the association between baseline HBsAg positivity and hospital-recorded tumor occurrence varied over time and was most pronounced during early follow-up. In age-stratified time-varying analyses, HBsAg positivity showed a broadly similar temporal pattern in both age groups (Figure S3).

### 3.4. Stratified Analysis

**Figure 5:**
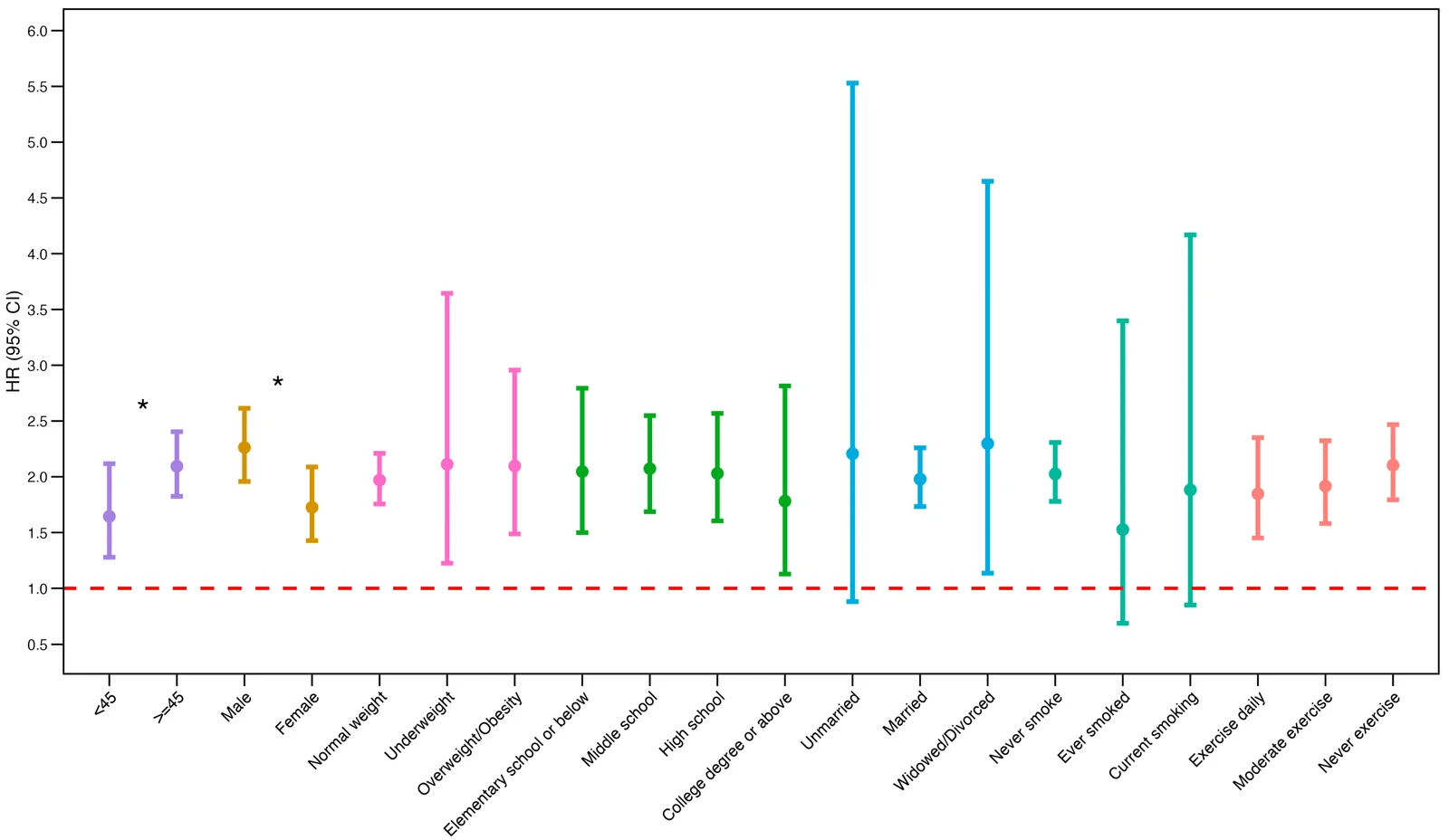
Stratified analysis of the association between HBsAg positivity and tumor risk. The horizontal dashed line indicates HR = 1. An asterisk (*) indicates a *p* value for interaction < 0.05.

Figure 5 showed that gender and age may influence the association between HBsAg exposure and tumor risk. We found that the risk effect of HBsAg(+) was higher in individuals aged ≥ 45 years old (HR, 2.09; 95% CI, 1.82–2.40) compared to younger populations (HR,1.64;95% CI,1.28–2.12). Moreover, the risk of tumor due to HBsAg exposure was higher in men (HR,2.26; 95% CI,1.96–2.61) than in women (HR,1.73; 95% CI,1.43–2.09) (Table S7). In contrast, we found no significant differences between the groups for other potential confounders, including BMI, alcohol consumption frequency, education level, ethnicity, exercise frequency, marital status, and smoking status (all *p* > 0.05).

### 3.5. Sensitivity Analysis Results

Sensitivity analyses yielded results consistent with the primary analysis. Estimates derived from the five imputed datasets were similar to those obtained from the main multivariable Cox model (Table S8). In the 1- and 2-year landmark analyses, the associations were attenuated but remained statistically significant (Table S9). After combining some covariates into categories, the results from Models 1–3 remained consistent (Figure S4). Moreover, the time-dependent analyses based on the imputed datasets showed only minor variations in the temporal risk pattern, without materially altering the overall conclusions (Figure S5).

## 4. Discussion

This 12-year prospective cohort study conducted in southern China included 538,573 participants. Our results showed a significant association between HBsAg(+) and tumor risk. Compared to the HBsAg(−) group, the pooled hazard ratios for all-site tumors, digestive system malignancies, and benign tumors in the HBsAg(+) group were 2.02 (95% CI, 1.79 –2.27), 4.28 (95% CI, 3.51–5.22), and 1.52 (95% CI, 1.27–1.82), respectively. Further analysis using a time-dependent Cox model revealed that the risk effect of HBsAg was most pronounced in the early follow-up period and gradually diminished over time. Stratified analysis showed that tumor risk was more significant in men and individuals aged ≥ 45 years. Various sensitivity analyses confirmed the robustness of the association between HBsAg(+) and tumor risk.

### 4.1. Impact of HBsAg Exposure on Tumor Risk

Baseline HBsAg positivity was associated with an increased risk of all-site tumors. Although a strong association was observed for digestive system malignancies, this category included liver and intrahepatic bile duct malignancies. Further subdivision showed that the association appeared to be largely driven by hepatic malignancies, particularly hepatocellular carcinoma, whereas no statistically significant association was observed for extrahepatic digestive system malignancies. This finding is consistent with the well-established association between HBV infection and hepatocellular carcinoma demonstrated in previous prospective studies [23,24]. Notably, prospective studies on overall benign tumors are scarce, with a few organ-specific studies suggesting that HBV infection is more prevalent in patients with benign breast disease compared to healthy individuals. In our study, we assessed both all-site and benign tumor outcomes within the same population. However, the findings related to benign tumors require further validation through independent cohorts.

The predominance of hepatic malignancies is biologically plausible because HBV primarily targets hepatocytes. Chronic HBV infection may promote hepatocarcinogenesis through chronic inflammation and oxidative stress, as well as through HBV DNA integration, genomic instability, altered gene expression, and epigenetic dysregulation [8,25,26]. Although HBV DNA fragments have been detected in extrahepatic tissues, including the stomach and pancreas, their causal relevance remains uncertain [27]. Because the present study measured HBsAg only at baseline and lacked information on infection duration, viral replication, HBV DNA levels, and antiviral treatment, these mechanisms could not be directly evaluated.

### 4.2. Time-Varying Association Between Baseline HBsAg Positivity and Tumor Risk

The association between baseline HBsAg positivity and tumor risk varied significantly over follow-up. The stronger association during early follow-up may reflect depletion of susceptible individuals, occult disease, or differential diagnostic surveillance [28]. Nevertheless, the principal findings persisted after excluding tumor events recorded within the first 1 and 2 years, and qualitatively similar temporal patterns were observed in both age groups. Previous studies of chronic HBV infection have linked higher viral replication and persistent infection to an increased risk of hepatocellular carcinoma, whereas virological suppression and HBsAg seroclearance are associated with a lower long-term risk [24,29,30]. These findings reinforce the importance of further clinical evaluation of HBsAg-positive individuals and guideline-based liver cancer surveillance when indicated. However, because infection duration and viral activity were unavailable, time since cohort entry should not be interpreted as the duration or stage of HBV infection.

### 4.3. Sex-Specific Differences in the Association Between HBsAg Positivity and Tumor Risk

Stratified analysis showed that HBsAg(+) was associated with a higher tumor risk in men. This finding is consistent with previous studies [31], which have observed a stronger association between HBsAg(+) and tumor risk in men [32,33,34], particularly for all-site tumors and various gastrointestinal malignancies. We speculated that this may be related to gender-based biological differences. Sex hormones and their receptor pathways can influence the transcription and replication of HBV. The androgen/androgen receptor axis may enhance viral-related transcription, while estrogen signaling may have an inhibitory effect, leading to a higher “background risk” [35,36,37] in males due to chronic antigenic stimulation and inflammation exposure. Additionally, the effect of sex hormones on tumor risk associated with HBsAg(+) may involve immune modulation, influencing immune surveillance and the tumor microenvironment, contributing to gender differences in risk [35,38]. Overall, our findings suggest that sex should be considered when evaluating tumor risk among HBsAg-positive individuals, which may help refine risk stratification and monitoring strategies.

### 4.4. Age-Specific Differences in the Association Between HBsAg Positivity and Tumor Risk

In addition, our study observed that the association between HBsAg(+) and tumor risk was more pronounced in individuals aged ≥45 years and relatively weaker in those aged <45 years, suggesting that age may have a modifying effect on the HBsAg-related tumor risk. Previous studies have shown that the tumor risk associated with HBV infection is more pronounced in middle-aged and older populations, likely due to the cumulative effects of viral exposure, age-related decline in immune surveillance, and prolonged low-grade chronic inflammation [39,40,41,42]. Additionally, several large-scale cohort studies have indicated that the risk of developing malignant tumors in HBV-infected individuals increases with age [9,34,43]. These findings are consistent with our results and highlight the need for focused tumor prevention, control, and follow-up management in middle-aged and older HBsAg(+) individuals.

### 4.5. Study Strengths and Limitations

Our study has several strengths. First, we used multiple imputation and propensity score matching to minimize the impact of missing data and confounding factors, enhancing the robustness of our results. Second, our study included participants from diverse demographic and behavioral backgrounds, thereby increasing the generalizability of our findings. Finally, the large sample size and long follow-up period provided sufficient statistical power to detect the effect of HBsAg(+) on the risk of all-site and site-specific tumors. Additionally, we employed a time-dependent Cox model to address violations of the proportional hazard assumption, revealing the dynamic changes in HBsAg-related tumor risk over time.

Nevertheless, some potential limitations should be noted. First, our study used baseline HBsAg status as a serological marker of HBV infection, without further distinguishing between viral replication levels, antiviral treatment, or serological outcomes. Our findings should thus be interpreted as associations with baseline HBsAg positivity. Second, tumor outcomes were ascertained through linkage to a hospital discharge database, while individual-level information on death, migration, and loss to follow-up was unavailable. Tumor events not captured by the linked database may also have been missed. Third, despite controlling for various confounders and achieving balance between groups using propensity score matching, unmeasured confounding may still be present. Fourth, missing data may affect our results, and we used multiple imputation to address missing covariates and performed multiple sensitivity analyses. Finally, this study did not include antiviral treatment data; we could not assess the potential impact of antiviral therapy on tumor risk in HBsAg(+) individuals. These issues require further confirmation in future studies.

## 5. Conclusions

In conclusion, baseline HBsAg positivity was associated with increased risks of hospital-recorded all-site tumors, benign tumors, and digestive system malignancies. The association with digestive system malignancies was primarily driven by liver and intrahepatic bile duct malignancies, particularly hepatocellular carcinoma, whereas no significant association was observed for extrahepatic digestive system malignancies. The associations varied over follow-up and were generally strongest during the early years after cohort entry. Stronger associations with all-site tumors were observed among men and participants aged ≥45 years. These findings support the potential value of baseline HBsAg status in tumor risk assessment and reinforce the importance of appropriate clinical evaluation and guideline-based liver cancer surveillance among HBsAg-positive individuals.

## Figures and Tables

**Table 1:** Baseline features of participants in the HBsAg-positive and HBsAg-negative groups before and after matching.

Characteristics	Before Matching			After Matching		
	HBsAg-Positive Group	HBsAg-Negative Group	*p* Value	HBsAg-Positive Group	HBsAg-Negative Group	*p* Value
Number of participants	11,703	526,870	-	11,701	11,701	-
Age (mean ± SD, year)	51.72 ± 16.04	48.20 ± 17.50	<0.001	51.72 ± 16.04	51.96 ± 16.75	0.27
Age Group			<0.001			0.36
<45 years	4269 (36.48)	237,580 (45.09)		4268 (36.48)	4200 (35.89)	
≥45 years	7434 (63.52)	289,290 (54.91)		7433 (63.52)	7501 (64.11)	
Gender (%)			<0.001			0.30
Female	5008 (42.79)	275,218 (52.24)		5008 (42.80)	5088 (43.48)	
Male	6695 (57.21)	251,652 (47.76)		6693 (57.20)	6613 (56.52)	
BMI (mean ± SD, kg/m^2^)	22.70 ± 2.92	22.03 ± 2.50	<0.001	22.69 ± 2.89	22.69 ± 2.81	0.97
Ethnicity (%)						1.00
Han	11,637 (99.44)	515,954 (97.93)		11,635 (99.44)	11,635 (99.44)	
Minority	66 (0.56)	10,916 (2.07)		66 (0.56)	66 (0.56)	
Smoking status (%)			<0.001			0.11
Never smoke	10,704 (91.46)	492,516 (93.48)		10,702 (91.46)	10,722 (91.63)	
Ever smoked	240 (2.05)	5981 (1.14)		240 (2.05)	275 (2.35)	
Current smoking	759 (6.49)	28,373 (5.39)		759 (6.49)	704 (6.02)	
Marital status (%)			<0.001			0.63
Unmarried	1012 (8.65)	95,084 (18.05)		1012 (8.65)	985 (8.42)	
Married	10,225 (87.37)	407,959 (77.43)		10,223 (87.37)	10,227 (87.40)	
Widowed/Divorced	466 (3.98)	23,827 (4.52)		466 (3.98)	489 (4.18)	
Education level (%)			<0.001			0.72
Elementary school or below	1528 (13.06)	62,573 (11.88)		1528 (13.06)	1569 (13.41)	
Middle school	3415 (29.18)	146,431 (27.79)		3415 (29.19)	3458 (29.55)	
High school	4222 (36.08)	196,093 (37.22)		4220 (36.07)	4166 (35.60)	
College degree or above	2538 (21.69)	121,773 (23.11)		2538 (21.69)	2508 (21.43)	
Exercise frequency (%)			<0.001			0.97
Exercise daily	2511 (21.46)	72,590 (13.78)		2511 (21.46)	2526 (21.59)	
Moderate exercise	2919 (24.94)	136,642 (25.93)		2918 (24.94)	2909 (24.86)	
Never exercise	6273 (53.60)	317,638 (60.29)		6272 (53.60)	6266 (53.55)	
Alcohol frequency (%)			<0.001			0.48
Never drink	11,025 (94.21)	502,220 (95.32)		11,024 (94.21)	11,032 (94.28)	
Occasional drinking	563 (4.81)	18,017 (3.42)		562 (4.80)	549 (4.69)	
Frequent drinking	73 (0.62)	5348 (1.02)		73 (0.62)	87 (0.74)	
Drink daily	42 (0.36)	1285 (0.24)		42 (0.36)	33 (0.28)	

BMI, body mass index; HBsAg, hepatitis B surface antigen; SD, standard deviation.

## Data Availability

The data underlying this article cannot be shared due to privacy restrictions related to individual health data. The study protocol is available upon reasonable request from the corresponding author. This study was reported in accordance with STROBE guidelines for cohort studies.

## Supplementary Materials

The following supporting information can be downloaded at: https://www.mdpi.com/article/10.3390/cancers18213445/s1, Supplementary Methods: Statistical analysis; Figure S1: Convergence trajectories of mean BMI across five imputation chains; Figure S2: Density distributions of observed and imputed BMI values; Figure S3: Age-stratified time-varying association between baseline HBsAg positivity and hospital-recorded all-site tumors; Figure S4: Sensitivity analysis of the associations between baseline HBsAg positivity and tumor outcomes using alternative covariate categorizations; Figure S5: Time-varying associations between baseline HBsAg positivity and tumor outcomes across five imputed datasets; Table S1: Comparison of participant characteristics between the full cohort and the complete-case dataset; Table S2: Schoenfeld proportional hazards assumption test results across five imputed datasets; Table S3: Comparison of linear and logarithmic specifications for the time-varying coefficient of HBsAg across five imputed datasets; Table S4: Administrative observation time and hospital-recorded tumor events by baseline HBsAg status; Table S5: Distribution of tumor types by baseline HBsAg status; Table S6: Association between baseline HBsAg status and hospital-recorded tumor occurrence; Table S7: Subgroup analyses of the association between baseline HBsAg positivity and all-site tumors; Table S8: Cox regression estimates from the five imputed datasets; Table S9: Landmark sensitivity analyses excluding tumor events recorded within the first 1 and 2 years of follow-up.

## Abbreviations

BMI, body mass index; CI, confidence interval; HBV, Hepatitis B virus; HBsAg, hepatitis B surface antigen; HR, hazard ratio; NAFLD, non-alcoholic fatty liver disease; PH, Proportional Hazards; PSM, propensity score matching; SMD, Standardized Mean Difference.
