# Supplementary material for: Association Between Hepatitis B Surface Antigen Positivity and Tumor Incidence: A Large Prospective Cohort Study in Southern China
Source: Cancers (Basel). 2026 Oct 25;18(21):3445. doi: 10.3390/cancers18213445 (PMC13761828; doi:10.3390/cancers18213445)
Supplement: Supplementary file 1 [file cancers-18-03445-s001.zip › cancers-4568768-supplementary.pdf]

Supplementary materials

Methods

Statistical analysis

To control for confounding, propensity score matching (PSM) was performed. Propensity scores were estimated using a logistic regression model including age, sex, BMI, marital status, ethnicity, education level, physical activity, smoking status, and alcohol consumption. A 1:1 nearest-neighbor matching without replacement was applied, with a caliper of 0.2 times the standard deviation of the logit of the propensity score. Covariate balance after matching was assessed using standardized mean differences (SMD), with SMD <0.1 indicating adequate balance.

The final time-dependent model was selected based on the AIC minimum criterion to describe the time-varying trend of HBsAg risk. The logarithmic time-dependent Cox model was selected based on AIC(Table S3).

Table S1. Comparison of participant characteristics between the full cohort and the complete-case dataset

Characteristics	After Imputation (N=538573)	Complete cases (N=382536)	Missing n(%)
Number of participants			
Age , mean ± sd, year	48.28±17.47	48.26±17.12	0(0)
Gender(%)			
Male	258347(47.97)	181491(47.44)	0(0)
Female	280226(52.03)	201045(52.56)	0(0)
Ethnicity(%)			162(0.03)
Han	527591(97.96)	379945(99.32)	
Minority	10982(2.04)	2591(0.68)	
BMI,(mean ±SD, kg/m^2)	22.05±2.51	22.07±2.50	59633(11.10)
Education level(%)			86689(16.10)
Elementary school or below	64101(11.90)	46900(12.26)	
Middle school	149846(27.82)	101737(26.60)	
High school	200315(37.19)	146415(38.27)	
College degree or above	124311(23.08)	87484(22.87)	
Marital status(%)			47992(8.91)
Unmarried	96096(17.84)	67333(17.60)	
Married	418184(77.65)	297395(77.74)	
Widowed/Divorced	24293(4.51)	17808(4.66)	
Smoking status(%)			19787(3.67)
Never smoke	503220(93.44)	352234(92.08)	
Ever smoked	6221(1.16)	5268(1.38)	
Current smoking	29132(5.41)	25034(6.54)	
Exercise frequency(%)			18804(3.49)
Exercise daily	75101(13.94)	60467(15.81)	
Moderate exercise	139561(25.91)	112863(29.50)	
Never exercise	323911(60.14)	209206(54.69)	
Alcohol frequency(%)			19647(3.65)
Never drink	513245(95.30)	361614(94.53)	
Occasional drinking	18580(3.45)	15392(4.02)	
Frequent drinking	5421(1.01)	4377(1.14)	
Drink daily	1327(0.25)	1153(0.30)	

SD, standard deviation.

Table S2. Schoenfeld proportional hazards assumption test results across five imputed datasets

Tumor type	Multiple imputation Dataset	Global Schoenfeld test *P* value
All-site tumors		
	MI1	<0.001
	MI2	<0.001
	MI3	<0.001
	MI4	<0.001
	MI5	<0.001
Digestive system malignancies		
	MI1	<0.001
	MI2	<0.001
	MI3	<0.001
	MI4	<0.001
	MI5	<0.001
Liver and intrahepatic bile duct malignancies		
	MI1	0.01
	MI2	0.002
	MI3	<0.001
	MI4	<0.001
	MI5	0.003
Hepatocellular carcinoma		
	MI1	0.04
	MI2	0.02
	MI3	0.01
	MI4	0.03
	MI5	0.07
Extrahepatic digestive system malignancies		
	MI1	0.03
	MI2	0.04
	MI3	0.03
	MI4	0.02
	MI5	0.06
Benign tumors		
	MI1	<0.001
	MI2	<0.001
	MI3	<0.001
	MI4	<0.001
	MI5	<0.001
Respiratory and intrathoracic malignancies		
	MI1	0.002

	MI2	0.001
	MI3	0.01
	MI4	<0.001
	MI5	0.02
Breast cancer		
	MI1	0.81
	MI2	0.30
	MI3	0.75
	MI4	0.83
	MI5	0.39
Thyroid and other endocrine gland malignancies		
	MI1	0.12
	MI2	0.07
	MI3	0.05
	MI4	0.30
	MI5	0.17

MI, multiple imputation.

Table S3. Comparison of linear and logarithmic specifications for the time-varying coefficient of HBsAg across five imputed datasets

Tumor type	Mean ΔAIC (range)	Selected time function
All-site tumors	26.18 (20.78–33.94)	Logarithmic
Digestive system malignancies	6.88 (4.40–8.96)	Logarithmic
Liver and intrahepatic bile duct cancer	2.22 (0.35–4.47)	Logarithmic
Extrahepatic digestive system cancers	2.41 (1.15–3.53)	Logarithmic
Benign tumors	4.96 (2.97–7.14)	Logarithmic
Respiratory and intrathoracic malignancies	3.10 (2.29–4.01)	Logarithmic

Note: ΔAIC was calculated as AIC for the linear time specification minus AIC for the logarithmic time specification; therefore, positive values favor the logarithmic specification. Models were fitted separately in each of the five imputed and propensity score–matched datasets. Within each dataset, the two specifications were compared using the same observations and covariate adjustment set. AIC values were not pooled using Rubin’s rules. AIC, Akaike information criterion.

Table S4. Administrative observation time and hospital-recorded tumor events by baseline HBsAg status

Baseline HBsAg status	Participants,n	Median years (IQR)	Total person-years	Tumor events
positive	11701	7.79 (7.79–8.78)	96534.17	1241
negative	11701	10.67 (9.20–11.27)	119114.05	797
Overall	23402	9.05 (7.79–10.84)	215648.22	2038

Data were derived from the first propensity score–matched imputed dataset. Administrative observation time was calculated from the baseline examination to the first hospital-recorded tumor diagnosis or December 30, 2022, whichever occurred first. Individual information on death and loss to follow-up was unavailable; therefore, the reported person-time represents administrative database observation. IQR, interquartile range; HBsAg, hepatitis B surface antigen.

Table S5. Distribution of tumor types by baseline HBsAg status

Tumor types	HBsAg-positive group (*n*,%)	HBsAg-negative group (*n*,%)	Total (n)
Digestive system malignancies	505(4.32)	151(1.29)	656
Benign tumors	315(2.69)	270(2.31)	585
Respiratory and intrathoracic malignancies	98(0.84)	101(0.86)	199
Other	103(0.88)	69(0.59)	172
Breast cancer	40(0.34)	43(0.37)	83
Thyroid and other endocrine gland malignancies	26(0.22)	39(0.33)	65
lymphatic, hematopoietic, and related tissues malignancies	33(0.28)	26(0.22)	59
female reproductive organs malignancies	39(0.33)	16(0.14)	55
male reproductive organs malignancies	21(0.18)	26(0.22)	47
urinary system malignancies	29(0.25)	15(0.13)	44
lips, oral cavity, and pharynx malignancies	19(0.16)	20(0.17)	39
Carcinoma in situ	13(0.11)	21(0.18)	34
Total (*n*)	1241(10.60)	797(6.81)	2038

Percentages represent the proportion of each tumor type within the respective group.

Table S6. Association between baseline HBsAg status and hospital-recorded tumor occurrence

HBsAg Status	Tumor Cases (*n*)	No Tumor (*n*)	Total (*n*)	RR（95%CI）	*P value*
Positive Group	1241	10460	11701	1.56	<0.001
Negative Group	797	10904	11701	(1.43,1.70)	

CI, Confidence Interval; *RR*, Risk Ratio.

Table S7. Subgroup analyses of the association between baseline HBsAg positivity and all-site tumors

Subgroup variable	Pooled HR(95%CI)	*P* for interaction
Age		0.04
≥45 years	2.09 (1.82–2.40)	
<45 years	1.64 (1.28–2.12)	
Gender		0.02
Female	1.73 (1.43–2.09)	
Male	2.26 (1.96–2.61)	
BMI		0.91
Normal weight	1.97 (1.76–2.21)	
Underweight	2.11 (1.22–3.64)	
Overweight/Obesity	2.10 (1.49–2.96)	
Education level		0.79
Elementary school or below	2.05 (1.50–2.79)	
Middle school	2.07 (1.69–2.55)	
High school	2.03 (1.60–2.57)	
College degree or above	1.78 (1.13–2.81)	
Marital status		0.88
Unmarried	2.21 (0.88–5.53)	
Married	1.98 (1.73–2.26)	
Widowed/Divorced	2.30 (1.14–4.65)	
Smoking status		0.88
Never smoke	2.03 (1.78–2.31)	
Ever smoked	1.53 (0.69–3.40)	
Current smoking	1.88 (0.85–4.17)	
Exercise frequency		0.56
Exercise daily	1.85 (1.45–2.35)	
Moderate exercise	1.92 (1.58–2.32)	
Never exercise	2.10 (1.79–2.47)	

HRs compare HBsAg-positive participants with HBsAg-negative participants. Estimates from the five imputed datasets were pooled using Rubin’s rules. Models were adjusted for the covariates included in Model 3, excluding the corresponding subgroup variable. The *P* value for interaction was calculated for each subgroup variable and is therefore presented only once for that variable. CI, Confidence Interval; HR, hazard ratio.

Table S8. Cox regression estimates from the five imputed datasets

Tumor type	MI Dataset	HR(95% CI)	*P* value
All-site tumors	MI1	2.10 (1.91-2.30)	<0.001
	MI2	2.05 (1.87-2.25)	<0.001
	MI3	2.03 (1.85-2.22)	<0.001
	MI4	1.97 (1.81-2.16)	<0.001
	MI5	1.93 (1.76-2.10)	<0.001
Digestive system malignancies	MI1	4.39 (3.65-5.29)	<0.001
	MI2	4.15 (3.46-4.97)	<0.001
	MI3	4.17 (3.48-5.00)	<0.001
	MI4	4.49 (3.73-5.40)	<0.001
	MI5	4.21 (3.51-5.05)	<0.001
Liver and intrahepatic bile duct malignancies	MI1	14.34 (10.11-20.34)	<0.001
	MI2	14.20 (10.05-20.06)	<0.001
	MI3	15.48 (10.81-22.18)	<0.001
	MI4	20.55 (13.66-30.90)	<0.001
	MI5	13.75 (9.78-19.34)	<0.001
Hepatocellular carcinoma	MI1	13.86 (8.92-21.53)	<0.001
	MI2	17.16 (10.58-27.82)	<0.001
	MI3	19.50 (11.70-32.48)	<0.001
	MI4	33.93 (17.39-66.22)	<0.001
	MI5	19.24 (11.56-32.03)	<0.001
Extrahepatic digestive system malignancies	MI1	1.33 (1.02-1.73)	0.04
	MI2	1.26 (0.97-1.63)	0.09
	MI3	1.21 (0.93-1.57)	0.15
	MI4	1.28 (0.98-1.66)	0.07
	MI5	1.27 (0.98-1.66)	0.07
Benign tumors	MI1	1.58 (1.34-1.87)	<0.001
	MI2	1.49 (1.26-1.75)	<0.001
	MI3	1.53 (1.30-1.81)	<0.001
	MI4	1.54 (1.30-1.81)	<0.001
	MI5	1.46 (1.24-1.71)	<0.001
Respiratory and intrathoracic malignancies	MI1	1.38 (1.03-1.83)	0.02
	MI2	1.13 (0.86-1.48)	0.40
	MI3	1.25 (0.94-1.65)	0.12
	MI4	1.19 (0.91-1.57)	0.21
	MI5	1.32 (0.99-1.75)	0.06
Breast cancer	MI1	1.22 (0.78-1.89)	0.38
	MI2	1.66 (1.04-2.66)	0.04
	MI3	1.12 (0.72-1.72)	0.62
	MI4	0.92 (0.61-1.39)	0.70
	MI5	1.00 (0.66-1.52)	0.98
Thyroid and other endocrine gland malignancies	MI1	1.92 (1.13-3.26)	0.02
	MI2	1.23 (0.77-1.98)	0.39
	MI3	1.55 (0.94-2.55)	0.09

	MI4	0.97 (0.61-1.54)	0.90
	MI5	1.09 (0.68-1.73)	0.72

CI, Confidence Interval; HR, hazard ratio; MI, multiple imputation.

Table S9. Landmark sensitivity analyses excluding tumor events recorded within the first 1 and 2 years of follow-up

Tumor type	Primary analysis		1-year landmark analysis		2-year landmark	
	Pooled HR (95% CI)	*P* value	Pooled HR (95% CI)	*P* value	Pooled HR (95% CI)	*P* value
All-site tumors	2.02 (1.79,2.27)	<0.001	1.96 (1.74,2.22)	<0.001	1.78 (1.58,2.01)	<0.001
Digestive system malignancies	4.28 (3.51,5.22)	<0.001	4.14 (3.39,5.05)	<0.001	3.72 (3.04,4.56)	<0.001
Liver and intrahepatic bile duct malignancies	15.49 (8.99,26.67)	<0.001	14.80 (8.58,25.54)	<0.001	13.29 (7.79,22.69)	<0.001
Hepatocellular carcinoma	19.78 (7.19,54.44)	<0.001	19.03 (6.90,52.47)	<0.001	17.18 (6.43,45.92)	<0.001
Extrahepatic digestive system malignancies	1.27 (0.97,1.67)	0.09	1.27 (0.97,1.67)	0.08	1.10 (0.83,1.47)	0.49
Benign tumors	1.52 (1.27,1.82)	<0.001	1.50 (1.25,1.81)	<0.001	1.41 (1.17,1.70)	<0.001
Respiratory and intrathoracic malignancies	1.25 (0.89,1.75)	0.19	1.22 (0.87,1.70)	0.25	1.08 (0.76,1.53)	0.68
Breast malignancies	1.16 (0.56,2.39)	0.67	1.14 (0.56,2.32)	0.70	1.09 (0.55,2.13)	0.80
Thyroid and other endocrine malignancies	1.31 (0.56,3.09)	0.50	1.31 (0.56,3.09)	0.50	1.30 (0.57,2.95)	0.50

Values for participants and events are presented as ranges across the five imputed and independently propensity score–matched datasets. HRs compare participants with baseline HBsAg positivity with those with baseline HBsAg negativity. Cox regression models were fitted separately in each dataset, and the log-HR estimates and their variances were pooled using Rubin's rules. All estimates were obtained from Model 3, adjusted for age, sex, body mass index, ethnicity, marital status, educational attainment, smoking status, alcohol consumption frequency, and exercise frequency. For each landmark analysis, only participants who remained event-free and within the database-defined risk set at the specified landmark were included, and follow-up commenced at the landmark. HR, hazard ratio; CI, confidence interval.

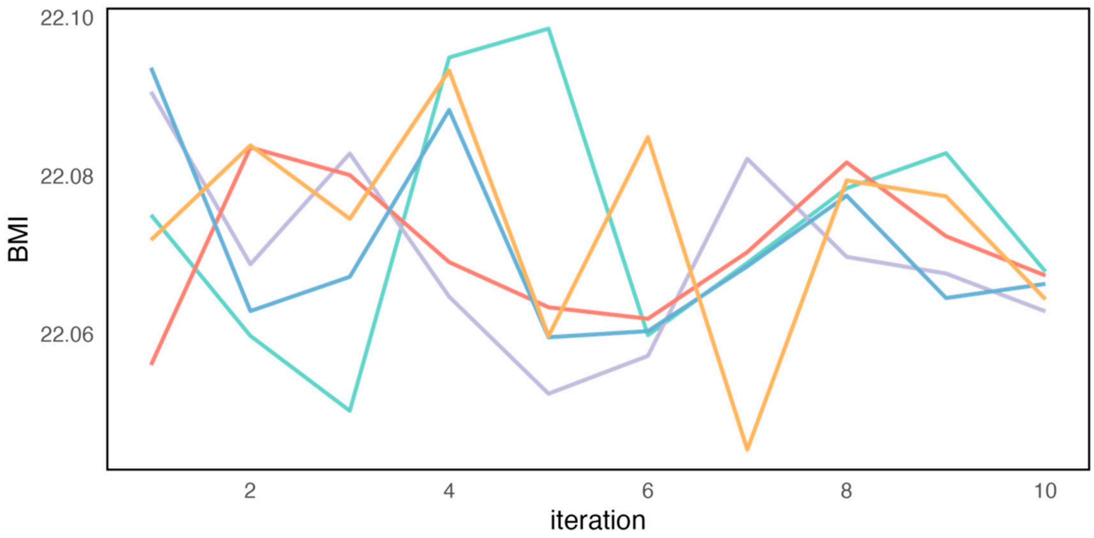

Figure S1. Convergence trajectories of mean BMI across five imputation chains

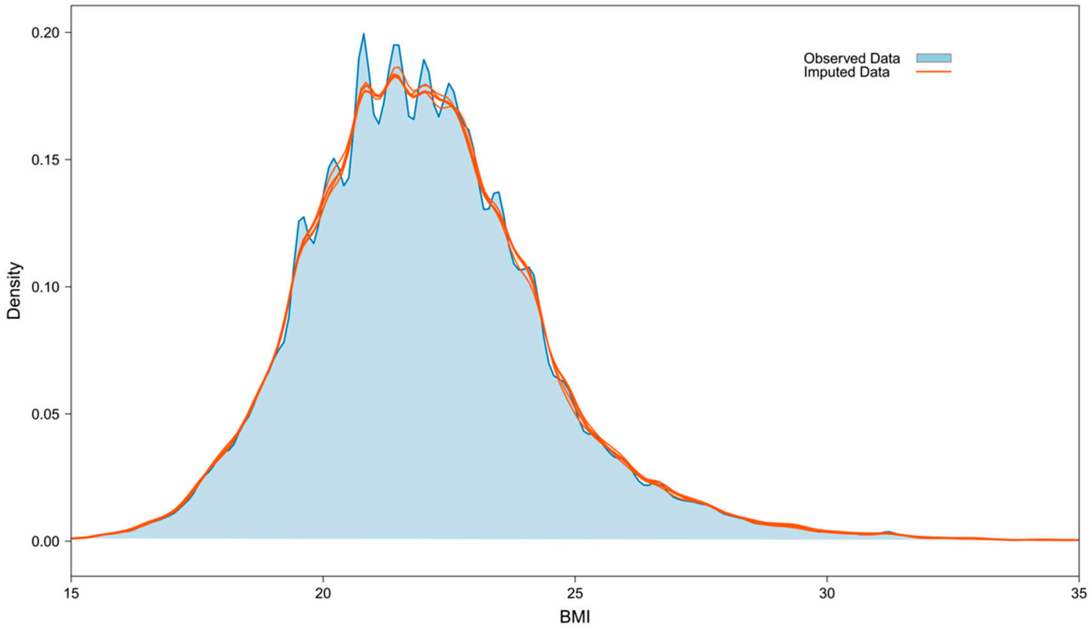

Figure S2. Density distributions of observed and imputed BMI values. The blue curve shows the distribution of observed values, while the orange curve represents the distribution of imputed values. The similar distribution patterns suggest that the imputed data closely match the original data, confirming the validity and stability of the multiple imputation results in this study.

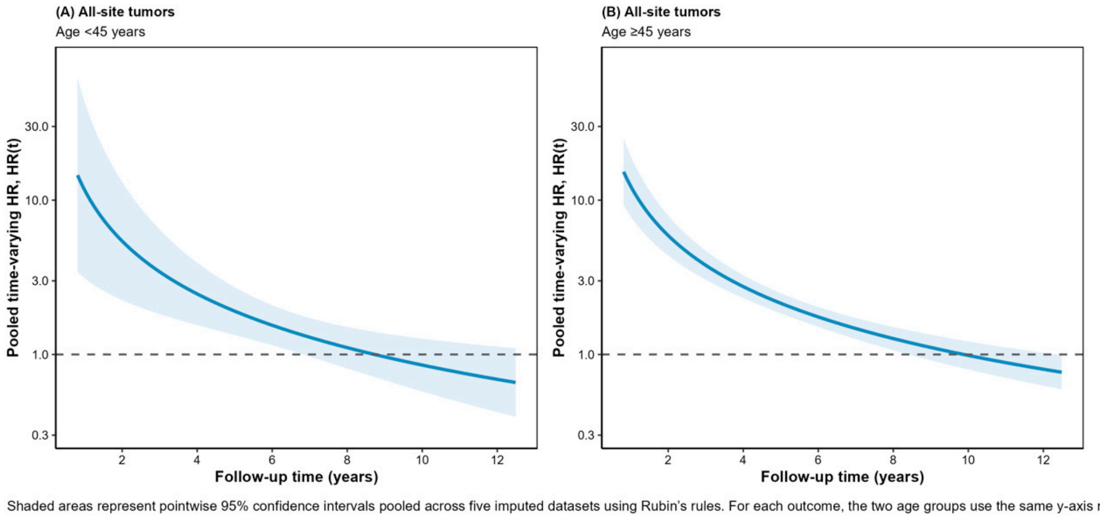

Figure S3. Age-stratified time-varying association between baseline HBsAg positivity and hospital-recorded all-site tumors. Time-varying hazard ratios (HRs) comparing HBsAg-positive with HBsAg-negative participants were estimated separately for participants aged <45 years (A) and ≥45 years (B). Solid blue lines represent pooled HR(t) estimates, and shaded areas represent pointwise 95% confidence intervals obtained across five imputed datasets using Rubin's rules. Models were adjusted for age within each stratum, sex, BMI, ethnicity, marital status, education level, exercise frequency, smoking status, and alcohol consumption. The horizontal dashed line indicates HR = 1. Both panels use the same logarithmic y-axis scale. HR, hazard ratio; CI, confidence interval.

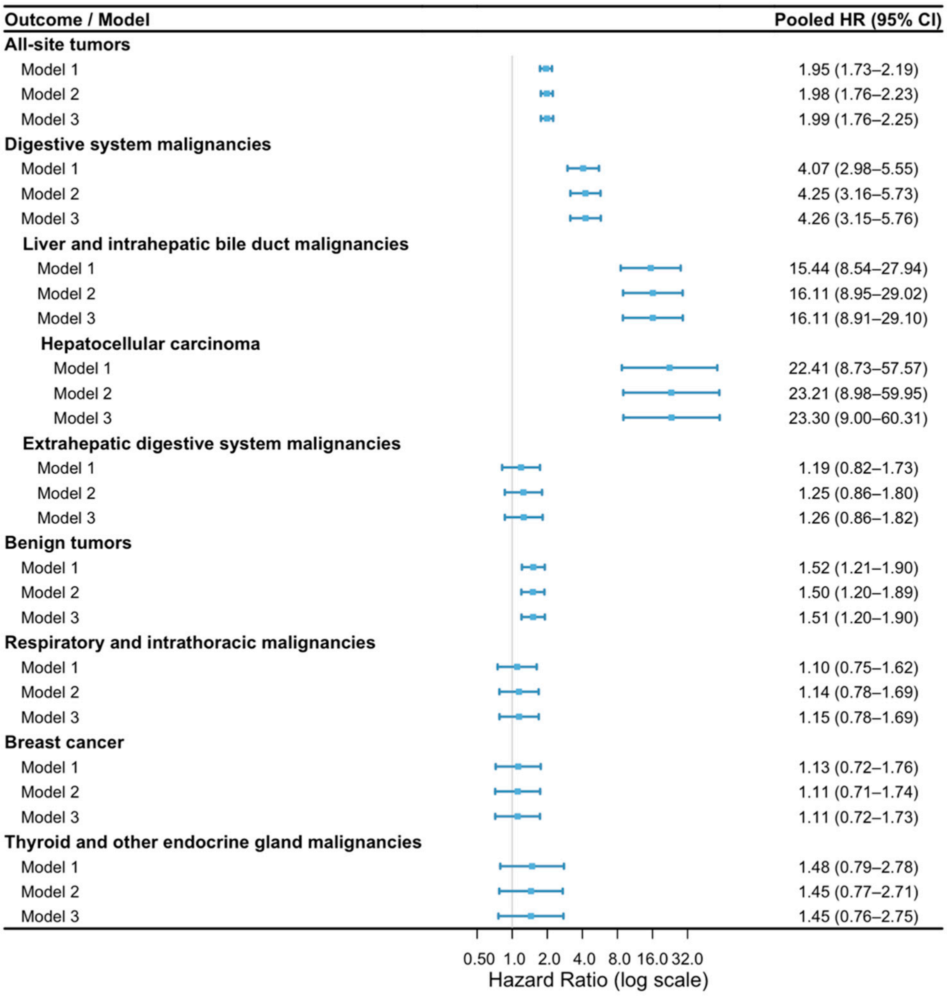

Figure S4. Sensitivity analysis of the associations between baseline HBsAg positivity and tumor outcomes using alternative covariate categorizations. In this analysis, smoking status was dichotomized as never versus ever smoking, and alcohol consumption was categorized as never, occasional, or regular. Model specifications were otherwise identical to those used in the primary analysis. Points and error bars represent pooled HRs and 95% CIs obtained from the five imputed datasets using Rubin’s rules.

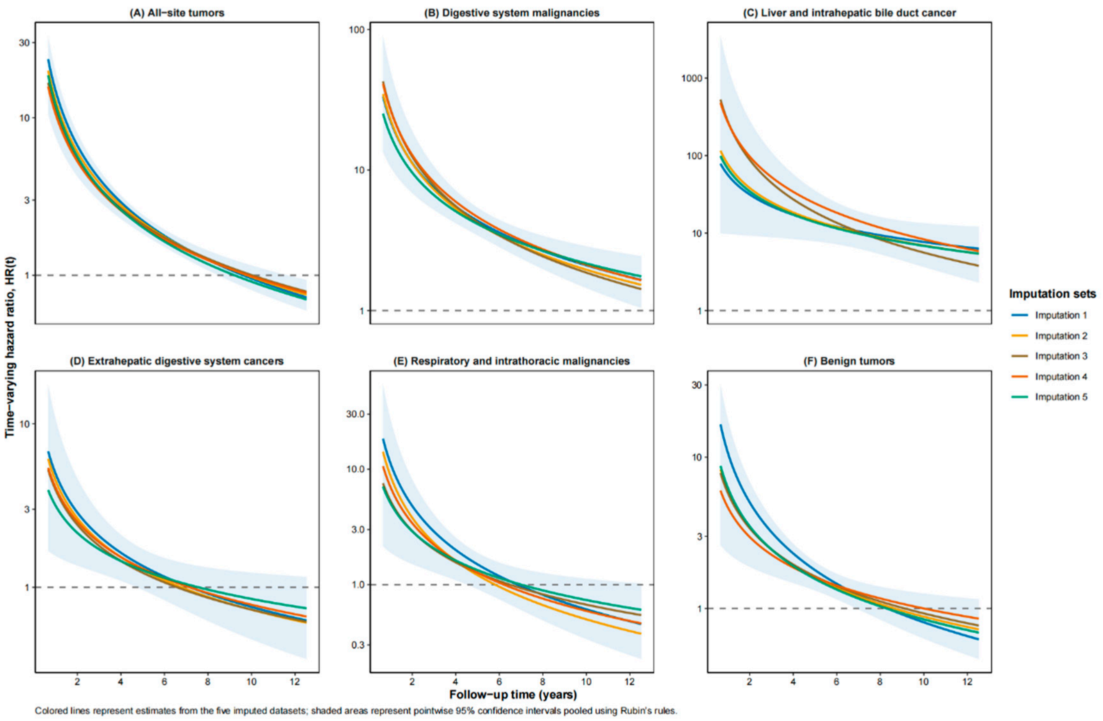

Figure S5. Time-varying associations between baseline HBsAg positivity and tumor outcomes across five imputed datasets. Multiple imputation by chained equations was used to generate five imputed datasets. When the proportional hazards assumption was violated, time-dependent Cox models were fitted across the imputed datasets for sensitivity analyses. Time-varying hazard ratios (95% Confidence Interval) for HBsAg and cancer outcomes were estimated, and the results were consistent with the primary analysis. The horizontal gray dashed line represents the reference line with HR = 1.
